# Spring Course

**Published:** 1872-04

**Authors:** 


### Spring Course.

The indications are that the Springs Course in the Ohio Dental College will be largely attended not only by students but by practitioners who wish to brush up a little, especially upon new methods and appliances.

Several have asked, " Will there be an examination, and will there be any credit toward an examination?" We endeavored to answer these questions definitely in the last REGISTER and in the circular sent out.

The object of this course is to give the greatest amount of thorough practical instruction in a short term and at the smallest expense. It is hardly possible that any one can come into this class and use the facilities that will be given and not learn that which will be of far more value to him than the expense involved. We shall hope to see a large representation of the profession in this session.

